# Case report: Stem cell-based suicide gene therapy mediated by the herpes simplex virus thymidine kinase gene reduces tumor progression in multifocal glioblastoma

**DOI:** 10.3389/fneur.2023.1060180

**Published:** 2023-03-22

**Authors:** Saeed Oraee-Yazdani, Mohammadhosein Akhlaghpasand, Fatemeh Rostami, Maryam Golmohammadi, Roozbeh Tavanaei, Gelareh Shokri, Maryam Hafizi, Maryam Oraee-Yazdani, Ali-Reza Zali, Masoud Soleimani

**Affiliations:** ^1^Functional Neurosurgery Research Center, Shohada Tajrish Comprehensive Neurosurgical Center of Excellence, Shahid Beheshti University of Medical Sciences, Tehran, Iran; ^2^Science and Research Branch, Islamic Azad University, Tehran, Iran; ^3^Stem Cell Technology Research Center, Tehran, Iran; ^4^Department of Research and Development, Sodour Ahrar Shargh Company, Tehran, Iran; ^5^Department of Hematology, Faculty of Medical Sciences, Tarbiat Modares University, Tehran, Iran

**Keywords:** multifocal glioblastoma, suicide gene therapy (SGT), stem cell gene therapy, HSV thymidine kinase, cell and gene therapies

## Abstract

**Introduction:**

The prognosis for glioblastoma multiforme (GBM), a malignant brain tumor, is poor despite recent advancements in treatments. Suicide gene therapy is a therapeutic strategy for cancer that requires a gene to encode a prodrug-activating enzyme which is then transduced into a vector, such as mesenchymal stem cells (MSCs). The vector is then injected into the tumor tissue and exerts its antitumor effects.

**Case presentation:**

A 37-year-old man presented to our department with two evident foci of glioblastoma multiforme at the left frontal and left parietal lobes. The patient received an injection of bone marrow-derived MSCs delivering the herpes simplex virus thymidine kinase (HSV-tk) gene to the frontal focus of the tumor, followed by ganciclovir administration as a prodrug for 14 days. For follow-up, the patient was periodically assessed using magnetic resonance imaging (MRI). The growth and recurrence patterns of the foci were assessed. After the injection on 09 February 2019, the patient's follow-up appointment on 19 December 2019 MRI revealed a recurrence of parietal focus. However, the frontal focus had a slight and unremarkable enhancement. On the last follow-up (18 March 2020), the left frontal focus had no prominent recurrence; however, the size of the left parietal focus increased and extended to the contralateral hemisphere through the corpus callosum. Eventually, the patient passed away on 16 July 2020 (progression-free survival (PFS) = 293 days, overall survival (OS) = 513 days).

**Conclusion:**

The gliomatous focus (frontal) treated with bone marrow-derived MSCs carrying the HSV-TK gene had a different pattern of growth and recurrence compared with the non-treated one (parietal).

**Trial registration:**

IRCT20200502047277N2. Registered 10 May 2020—Retrospectively registered, https://eng.irct.ir/trial/48110.

## Introduction

Glioblastoma multiforme (GBM) is the most common type of brain tumor malignancy in adults, and even with the best available therapy, the average survival time is < 12 months (1). The incidence rate of multiple synchronous GBM lesions is between 0.5 and 35%, with an average incidence rate of ~10% (2).

According to prior clinical trials, one of the most widely evaluated and encouraging therapeutic strategies for cancer is suicide gene therapy (3). In previous studies on glioma, the suicide gene therapy using the HSV-tk gene, which phosphorylates nucleoside analogs such as ganciclovir (GCV) to suppress DNA replication of cells, has been frequently investigated (4).

Efficient gene delivery to the target cancer cells is one of the significant issues in gene therapy (5). In addition to viral vectors, applying MSCs as gene delivery vehicles is unique in cancer treatment (6). This potential use of MSCs mainly originates from their significant capability to migrate into inflammatory sites and tumor microenvironments. They could also exert antitumor and proapoptotic effects on tumor cells (5). However, some studies have revealed that the immunosuppressive influence of MSCs could lead to cancerous transformation, tumor growth, and metastasis enhancement in experimental models. Owing to these findings, the use of MSCs in cancer has become a matter of debate, indicating the necessity of clinical trials to provide more evidence about their safety and efficacy (6, 7).

In this study, we report the case of a patient with multifocal glioblastoma multiforme with two gliomatous foci, one in the left frontal lobe and one in the left parietal lobe. Following the resection of the patient's left parietal focus, we treated another lesion in it with the MSC-HSV-tk/GCV approach. To the best of our knowledge, this is the first case report investigating the recurrence and growth patterns of the treated gliomatous foci with an MSC-HSV-tk/GCV approach compared with the control gliomatous foci in one patient.

## Case presentation

**Figure 1:**
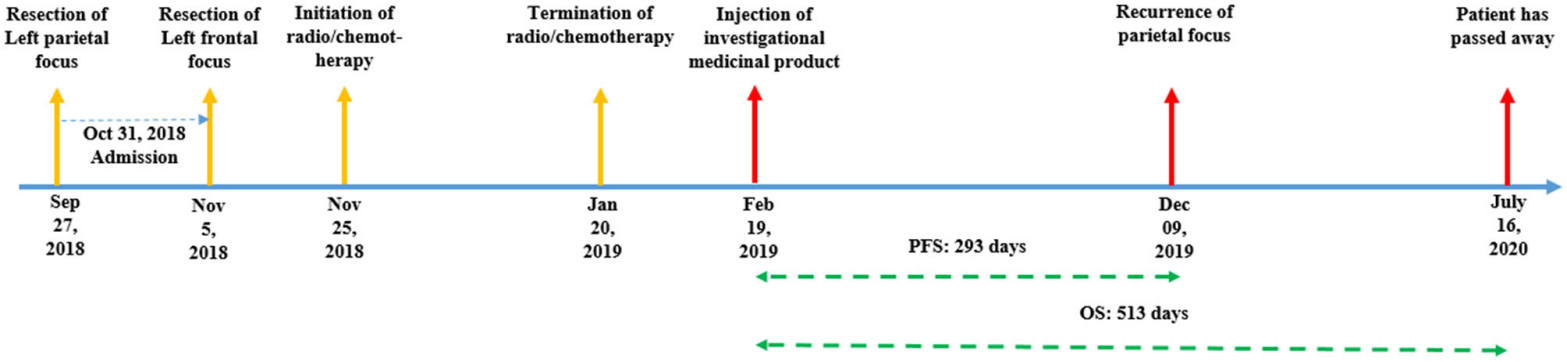
Timeline of the case presentation.

A 37-year-old man with multifocal glioblastoma multiforme and a Karnofsky Performance Score (KPS) of 90 was referred to the outpatient neurosurgery clinic of our hospital on 31 October 2018. He had two gliomatous foci in the left frontal and left parietal lobes. The patient had undergone surgical resection for his left parietal focus at another center 35 days before his presentation to our institution. He also underwent surgical resection at our institution for another lesion in the frontal lobe through a frontal craniotomy on 05 November 2018. He experienced episodes of focal seizures, which were controlled with levetiracetam 500 mg two times daily. The patient also reported having a paternal history of hypertension and diabetes mellitus type II. Our standard physical examinations showed a reduction in the muscle forces of the upper extremities (4/5 on both the left and right sides). Histopathological analysis of both foci revealed that these tumors were grade IV astrocytomas (glioblastoma multiforme). According to the immunohistochemistry and PCR tests, both foci were IDH1/2-wildtype, and the MGMT promoter was methylated under 5%. Standard radiation therapy was started on 25 November 2018, following which temozolomide (75 mg/m^2^/day) was taken 1 h before the initiation of radiation therapy for the first week and then continued separately for 54 days until 20 January 2019 (Figure 1). After the radio/chemotherapy was completed, the patient volunteered for participation in our stem cell-mediated gene therapy program for glioblastoma.

Our study was approved by the Ethics Committee of Medical Research at the Shahid Beheshti University of Medical Sciences (License number: IR.SBMU.REC.1400.002). For all procedures performed in this study, the patient was informed about the experimental nature of the treatment, unexpected outcomes, and possible adverse events, and then, written informed consent was obtained.

Autologous bone marrow-derived MSCs were collected from the iliac bone in the operation room under sterile conditions. After the aspiration, 100 ml of bone marrow blood sample was diluted in Hanks' Balanced Salt Solution (HBSS, Sigma) at a ratio of 1:1. The patient was discharged a day after the procedure. Utilizing the protocol discussed in our previous study (8), samples went through a density gradient centrifugation by Ficol (density 1.077 g.L^−1^, Sigma) at a ratio of 1:3. Samples were then centrifuged, and their mononuclear cell layer was recovered from the gradient interface. To isolate mononuclear cells, the cells were centrifuged three times, with less gradient and reduced time for separating platelets. To characterize the isolated cells, they were resuspended in PBS, and they were incubated and conjugated with monoclonal antibodies containing CD73, CD45, CD44, CD90, and CD105 (eBioScience, Inc., San Diego, CA) and different dyes subsequently. Finally, these markers were determined using FACScan flow cytometry (PartecPAS III-Partec, Germany). The culture medium was then changed every 3 days till the cell number reached 5 × 10^5^.

The Human Embryonic Kidney (HEK) 293 cell lines were obtained from the Iranian Biological Resource Center. Subsequently, these cell lines were cultured in high-glucose Dulbecco's Modified Eagle Medium (DMEM, Gibco, USA). This medium contains 10% fetal bovine serum (FBS, Gibco, USA), 1% non-essential amino acids (Invitrogen, USA), and L-glutamine (2 mM, Gibco, USA). The cells were cultured under the standard conditions of 95% humidity and 5% CO_2_ at 37°C.

**Figure 2:**
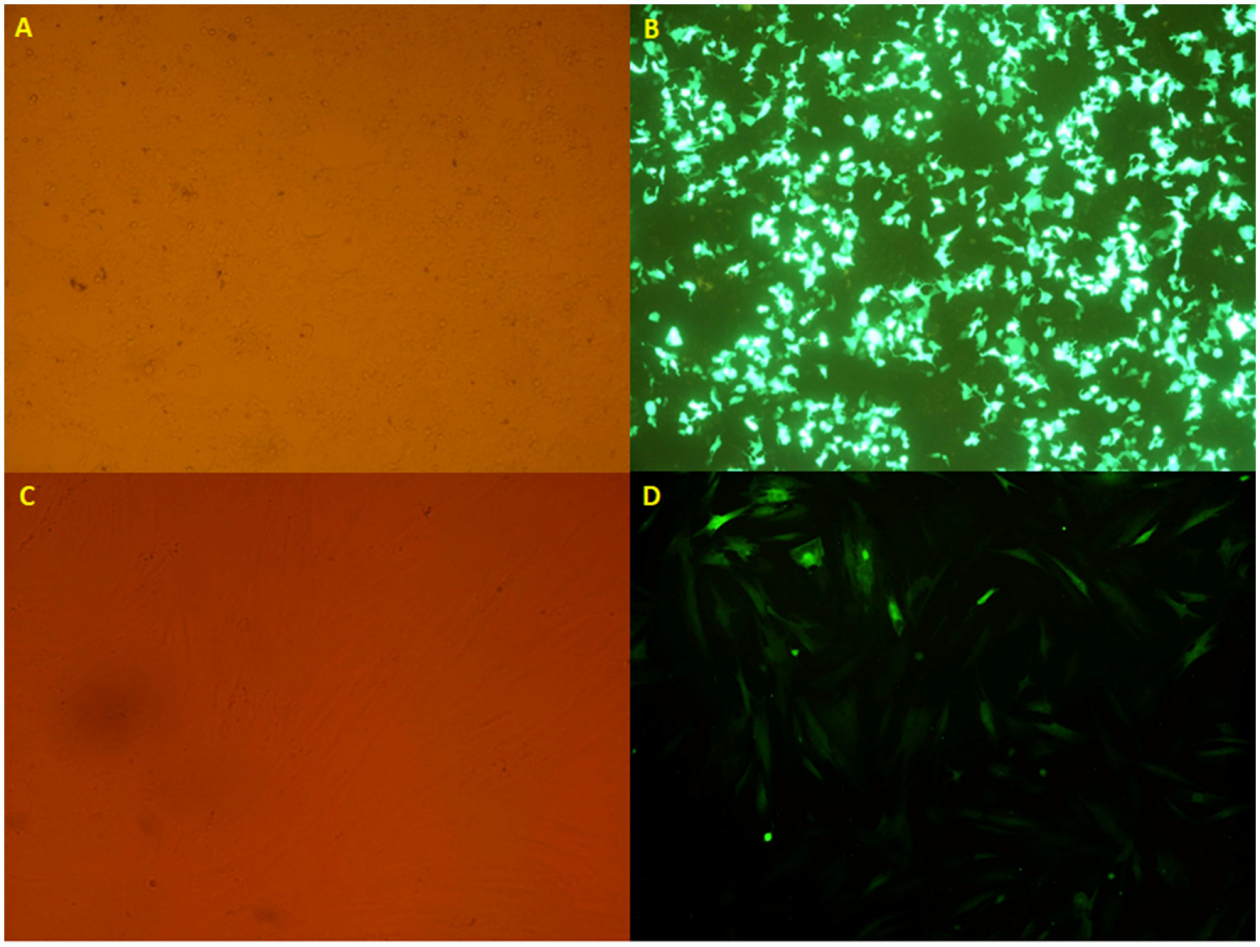
**(A, B)** The HEK 293T cell lines transfected with the calcium phosphate method, taken from an inverted microscope (10×), which shows **(A)** visible light and **(B)** fluorescent light. **(C, D)** The transduced mesenchymal stem cells originated from an inverted microscope (10×), which indicates **(C)** visible light and **(D)** fluorescent light.

The thymidine kinase enzyme was cloned into the pCDH-CMV-MCS-EF1-copGFP plasmid (Stem Cell Research Center, Iran) that was previously digested with BamHI and EcoRI endonuclease enzymes (Thermo Fisher, USA). For lentivirus production, HEK-293T cells were co-transfected with pCDH-thymidine kinase, pSPAX2 plasmid (packaging plasmid contains Gag, Pol, Rev, and Tat), and pMD2 plasmid [containing glycoprotein (G) of vesicular stomatitis viruses (VSVs)] by calcium phosphate reagent (Sigma Aldrich, USA) (Figure 2). The supernatants were harvested every 12 h for 3 days after transfection and concentrated by ultracentrifugation at 47,000 × g for 2 h at 4°C. To express thymidine kinase in MSCs, these cells were cultured in DMEM/F12 (Gibco, USA) medium and then transduced with pCDH-thymidine kinase lentivirus. A total of 3 × 10^5^ mesenchymal stem cells (MSCs) were seeded in a T25 flask and subsequently transduced the next day by considering the multiplicity of infection (MOI) of 40 (TU/cell) after determination *via* the serial dilution transduction method. In addition, transduced cells with pCDH-TK were selected by puromycin (Sigma Aldrich, USA) (2 μg/ml) as a eukaryotic selective marker. Following the purification of transduced cells using puromycin, genetically modified MSCs were washed three times with normal saline (Iranian Parenteral and Pharmaceutical Company, Iran).

**Figure 3:**
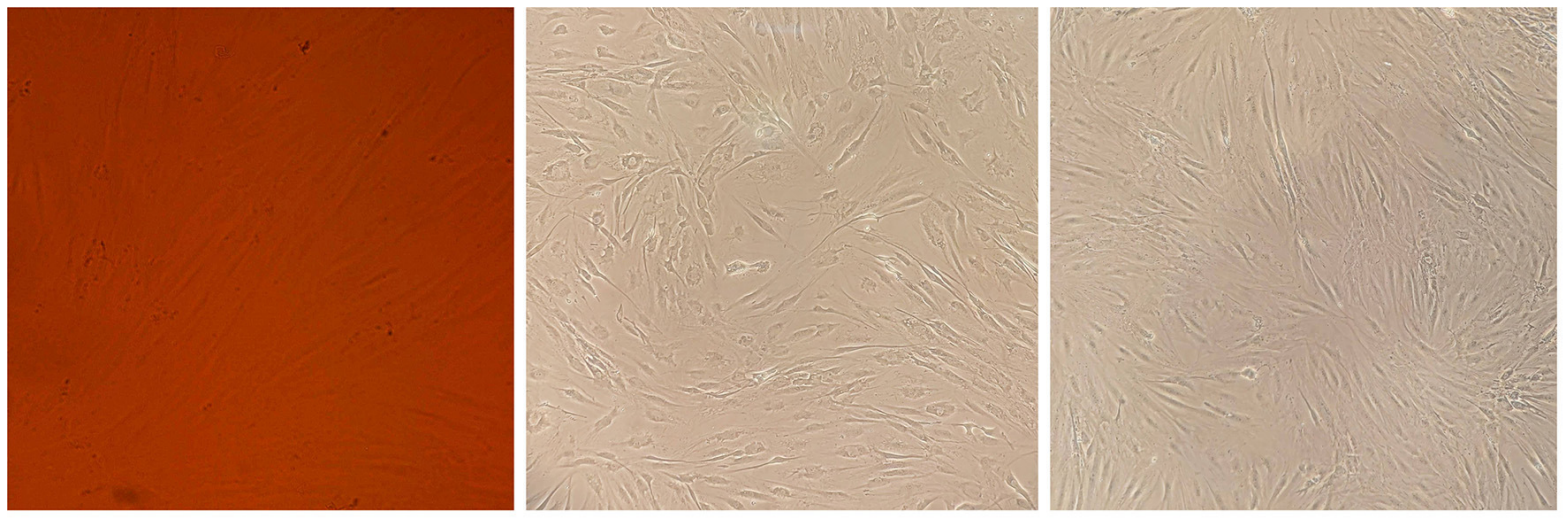
Cell morphology assay of MSCs derived from the patient's bone marrow. The spindle-shaped MSCs are visible at several growth rates.

**Figure 4:**
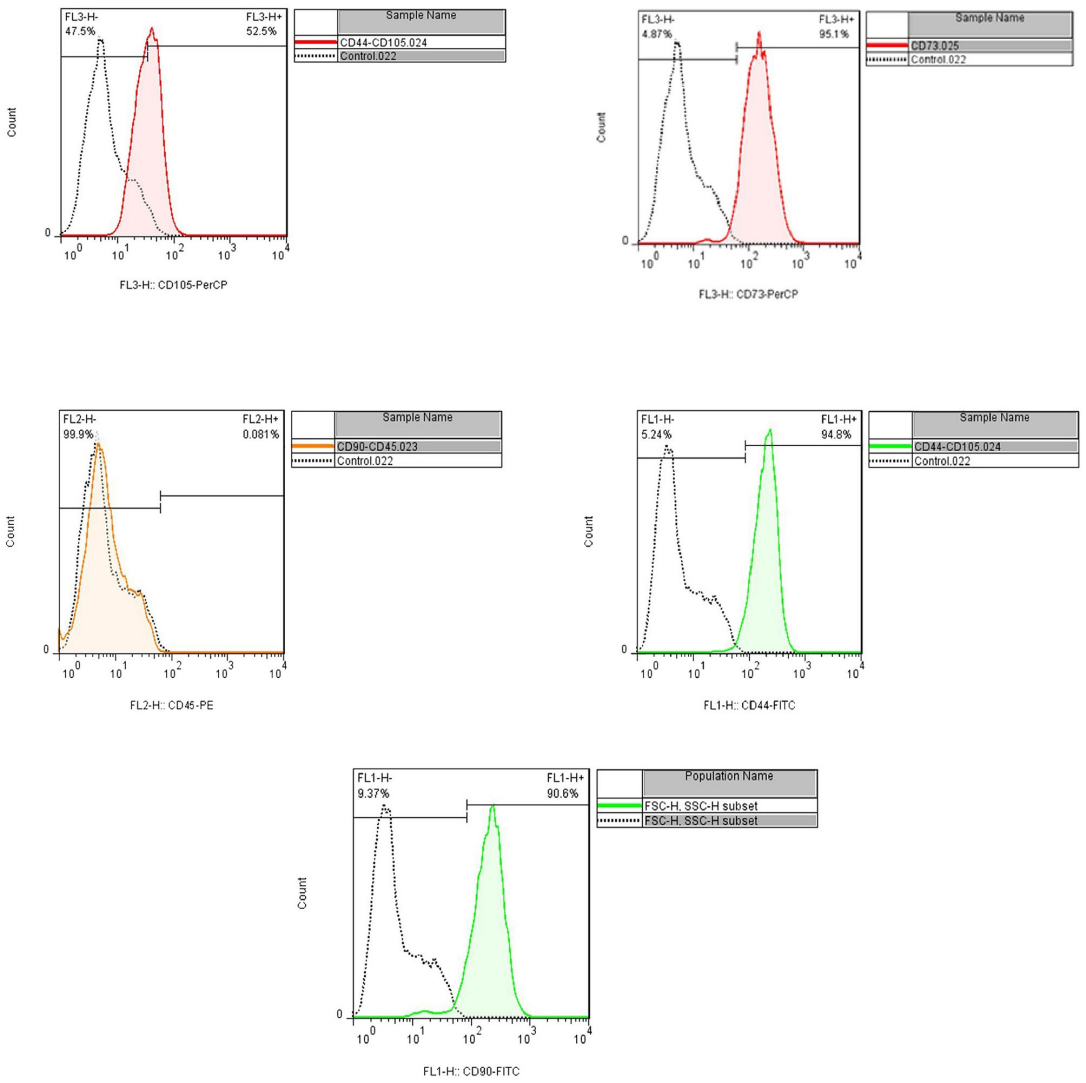
Flow cytometry assay indicated the positive expression of CD44, CD90, CD73, and CD105 (94.8, 90.6, 95.1, and 52.5%, respectively), and CD45 were not expressed (<0.08%).

Flow cytometry and cell morphology assay confirmed the characteristics of isolated cells from the patient as MSCs. The cell population indicated the positive expression of CD44, CD90, CD73, and CD105 (94.8, 90.6, 95.1, and 52.5%, respectively); however, CD45 were not expressed (< 0.08%) (Figures 3, 4).

After virus production and concentration, the virus titration was determined by using flow cytometry. Following the titration of recombinant viruses, the mesenchymal stem cells were transduced with a certain amount of the virus (MOI 40) (Figure 2).

The patient was hospitalized a day before surgery. Under general anesthesia, through a new burr hole, an injection catheter was placed in the bed of the frontal tumor focus with the guidance of a direct intraoperative navigation system (using a 1.5T MR system, Siemens Magnetom Trio, Siemens Medical Systems, Erlangen, Germany, and Synergy Cranial Version 2.2, Medtronic, Louisville, Colorado). The patient then received 5 × 10^5^ bone marrow-derived MSCs transfected by lentivirus containing HSV-tk enzyme in 1 cc volume *via* the injection catheter. The catheter was placed at the injection site for 1 min to prevent leakage. We administered 5 mg/kg of ganciclovir over 1 h and 48 h after cell injection. Ganciclovir was administered two times daily for 14 days (28 doses in total).

During the therapy, the patient was assessed for any adverse events based on the common terminology criteria for adverse events (CTCAE, version 4.3). Considering the adverse events of ganciclovir, blood and urine samples from the patient were obtained and analyzed regularly during hospitalization to assess hematologic toxicity and renal impairment.

The patient was followed up for tumor recurrence and progression using magnetic resonance imaging every 3 months. The MRI images were compared with those taken before the injection and checked by two independent neurosurgeons. The Response Evaluation Criteria in Solid Tumors (RECIST) was used for MRI assessments. In this criteria, recurrence or progression of the tumor was defined as having at least a 20% increase in the sum of the longest diameters of the target lesions (9).

After the cell injection, our patient was in a good general state and was conscious. The patient had a previous history of seizures, and because the regular seizure medication was discontinued, he experienced a seizure episode 3 months after gene therapy. Generally, adverse events and systemic complications were not observed.

**Figure 5:**
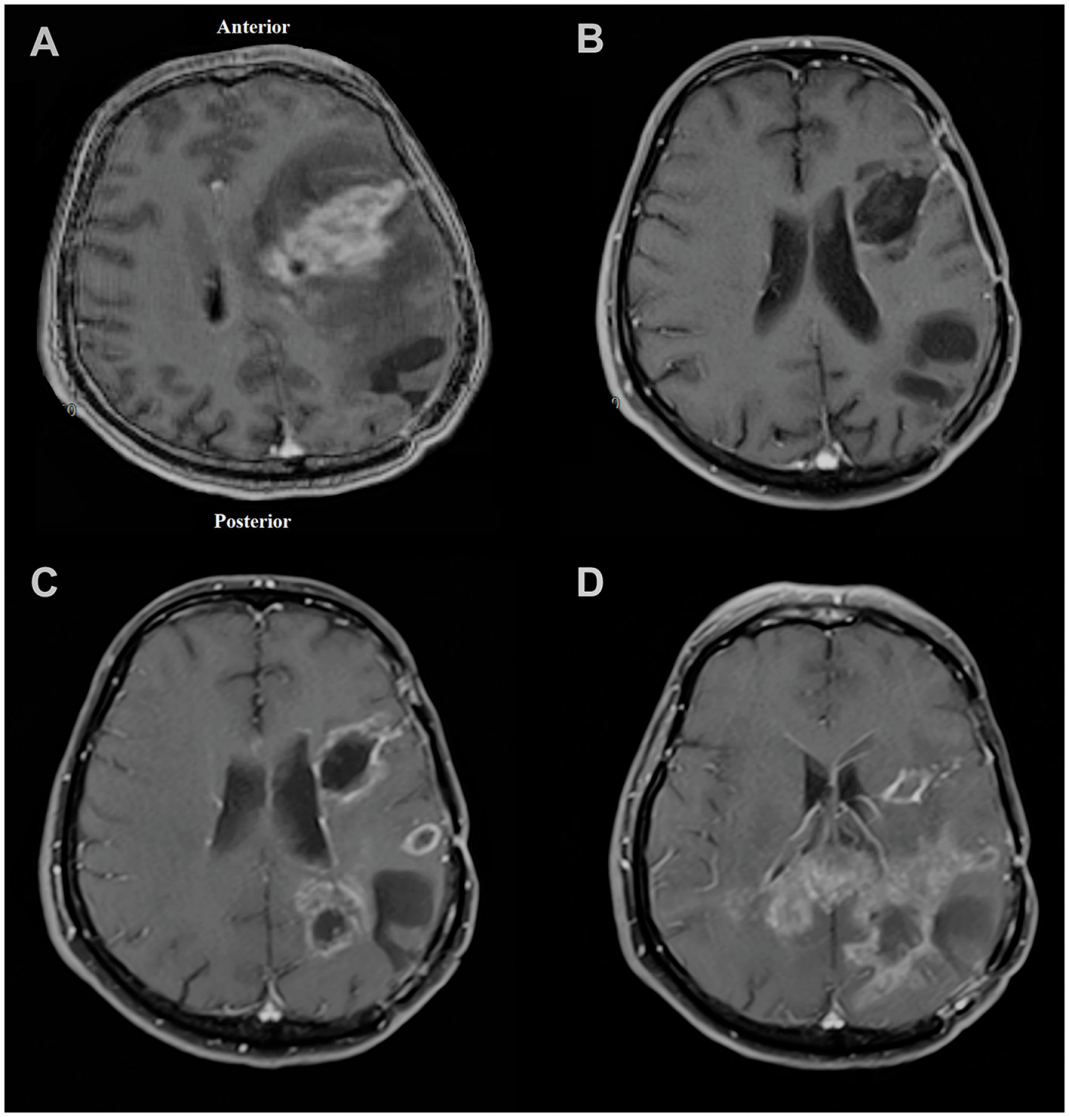
Magnetic resonance imaging of the patient in **(A)** November 2018 showing left frontal lesion and evidence of craniotomy related to previous surgery for parietal lesion 1 month earlier; **(B)** February 2019 with no obvious evidence of recurrence at the time of injection to the frontal focus; **(C)** December 2019 showing first evidence of recurrence of tumor mostly at the parietal site; **(D)** March 2020 showing left frontal focus had no prominent recurrence; however, the size of the left parietal focus increased and extended to the contralateral hemisphere through the corpus callosum.

After the injection on 19 February 2019, the patient's follow-up revealed a recurrence of the parietal focus on 09 December 2019. However, the frontal focus had a slight and unremarkable enhancement. On the last radiological follow-up (on 18 March 2020), the frontal focus showed no prominent recurrence; however, the size of the parietal focus increased and extended to the contralateral hemisphere through the corpus callosum (Figure 5). Eventually, the patient passed away on 16 July 2020 (progression-free survival (PFS) = 293 days, overall survival (OS) = 513 days) (Figure 1).

## Discussion

Regarding the results of suicide gene therapy in malignant glioma, it has been suggested that suitable results depend on promoting the delivery system (10, 11). Therefore, we decided to improve the delivery system in suicide gene therapy by using MSCs and lentiviral vectors. Furthermore, we attempted to reduce the effects of confounding factors to the extent possible by comparing two tumor foci with different treatment courses in a single patient. This type of comparison attenuates the power of factors and confounders that affect the result of treatments in GBM clinical trials, such as age, sex, Karnofsky score, and genome of the patients (12).

Retroviral and adenoviral vectors were the first generation of vehicles used for clinical suicide gene therapy in glioblastoma multiforme. Early investigations revealed that these vehicles are safe and could have relative efficacy in treating both primary and recurrent glioblastoma (13–16). However, after conducting larger trials, this possible efficacy was questioned. In 2000, Rainov and his colleagues conducted the first phase III, multicenter, randomized clinical study on 248 patients, demonstrating an insignificant effect of suicide gene therapy based on viral vectors on primary glioblastoma (16). Then, another phase III clinical trial indicated that adenovirus-mediated gene therapy with TMK could increase the time to death and reintervention time in patients with primary glioblastoma but could not significantly change the OS (17). Despite these results, Nan Ji and colleagues indicated that the use of viral-mediated suicide gene therapy in recurrent glioblastoma has a remarkable effect on the OS of these patients (18).

In some experimental studies, the efficacy of MSCs in delivering genes to various tumor models such as GBM, non-small-cell lung cancer, and breast cancer has been suggested (17, 19–22). However, clinical studies are limited to advanced gastrointestinal tumors and GBM (8, 22). Primarily due to safety concerns, the scope of such studies is limited. Einem et al. were the first group to utilize genetically engineered MSCs in a clinical setting. Their study was a single-arm phase I/II clinical trial to evaluate the safety and efficacy of using genetically modified autologous MSCs as delivery vehicles for cell-based gene therapy in the treatment of advanced, recurrent, or metastatic gastrointestinal or hepatopancreatobiliary adenocarcinoma (TREAT-ME1 study). In the study's first phase, six patients received this therapy; the results showed that this treatment was safe during three cycles with different concentrations. In the second phase, 16 patients were divided into two groups, including patients with GI adenocarcinoma who were qualified for surgery with prior neoadjuvant treatment and advanced adenocarcinoma cases with recurrence or progression (22, 23).

The rationality of using MSC as a gene delivery vehicle originates from the underlying molecular mechanism that causes tropism of the MSCs toward the tumor microenvironment (24–26). One of the most important factors constituting this tendency is the chemotactic gradient of secreted factors released from cancer cells, such as epidermal growth factor (EGF), fibroblast growth factor (FGF), platelet-derived growth factor (PDGF), vascular endothelial growth factor A (VEGF-A), stromal cell-derived factor-1 (SDF-1), hepatocyte growth factor (HGF), and transforming growth factor (TGF)-β, granulocyte colony-stimulating factor (G-CSF), granulocyte-macrophage colony-stimulating factor (GM-CSF), monocyte chemoattractant protein-1 (MCP-1), IL-8, IL-6, and urokinase-type plasminogen activator (24–30). However, recent findings have suggested that vascular adhesion molecules (VCAM-1) and very late antigen-4 (VLA-4) play a pivotal role in MSC adhesion. In fact, it has been shown that TNF-a induces VCAM-1 by activating the NF-κB signaling pathway and thereby results in cell accumulation (31).

In addition to homing properties, MSC gene delivery vehicles could exert antitumor and proapoptotic effects (32). Prior studies have shown that MSCs could cease the proliferation of hepatoma, lymphoma, and insulinoma cells and arrest their cell cycle at G0/G1. Furthermore, they could stimulate apoptosis in cancer cells by blocking Akt and NF-κB signaling pathways (7, 33). MSCs also inhibit the Wnt and AKT signaling pathways leading to tumor growth inhibition (34–36) and exert dose-dependent apoptosis of capillaries with inhibition of angiogenesis *via* Cx43 expression (37). Moreover, they reduce tumor growth through the attraction of granulocytes and macrophages, which leads to the production of chemokines (like IP-10 and IL-8) and an increase in the homing of activated lymphocytes (38). These findings also have been supported by investigations in other cancer models. A local injection of MSCs prevented tumor growth and increased survival time in the rat model of glioma. Furthermore, both systemic and local injections of MSCs resulted in attenuation of growth and metastasis in breast cancer models (39, 40).

Despite the low distribution of ganciclovir in the CNS, clinical studies have indicated the therapeutic effect of the HSV-tk/GCV approach on GBM (14, 18, 41). This could be addressed by the loss of integrity in the blood–brain barrier (BBB) and the development of a new vasculature structure known as the blood–tumor barrier (BTB). In fact, BTB does not have the uniformity and integrity of BBB, which leads to increased permeability and active efflux of molecules through it (42, 43). Accordingly, drug distribution in tumor tissue follows different patterns compared with normal CNS tissue. The results of pharmacokinetic studies, such as the one investigating the targeted therapy of “dasatanib” in glioma, support the aforementioned rationale (44, 45). Thus, it is belived that ganciclovir would have a therapeutic effect on the CNS.

There are some limitations associated with investigating the use of stem cell-based suicide gene therapy in multifocal glioblastoma. Some characteristics of stem cells, such as their immunosuppressive and proangiogenic properties as well as the secretion of trophic factors, still make their safe application in tumor treatment questionable (32, 46). Thus, these cells should be used carefully and in a limited number of patients who should be followed up closely to ensure safety. After the recurrence of the parietal tumor focus, the patient's condition was not suitable for resection. Hence, we did not inject mesenchymal stem cells containing the HSV-tk gene into the tumor tissue without prior resection as it was risky.

## Conclusion

According to study results, a treatment strategy utilizing bone marrow-derived MSCs delivering the HSV-tk gene is feasible in multifocal glioblastoma and is safe for further investigations. Although this case report indicated the attenuated growth of a glioblastoma focus treated with MSCs containing the HSV-tk gene, the safety and effectiveness of this intervention require more extensive investigations.

## Data availability statement

The original contributions presented in the study are included in the article/supplementary material, further inquiries can be directed to the corresponding author.

## Ethics statement

The studies involving human participants were reviewed and approved by the Research Ethics Committees of Shahid Beheshti University of Medical Sciences. Written informed consent to participate in this study was provided by the patients/participants or patients/participants' legal guardian/next of kin. Written informed consent was obtained from the individual(s) and/or minor(s)' legal guardian/next of kin for the publication of any potentially identifiable images or data included in this article.

## Author contributions

SO-Y: project designing and conducting clinical procedures. MA: writing the manuscript and performing a literature review. FR, GS, and MH: conducting the laboratory procedures. MG: editing the manuscript and designing the follow-up. RT: editing the manuscript and conducting the laboratory procedures. MO-Y: following up with the patients. A-RZ: supervising the clinical procedures. MS: supervising the laboratory procedures. All authors contributed to the article and approved the submitted version.
